# Impact of preprocedural anticoagulation on the incidence of silent cerebral embolisms after catheter ablation of atrial fibrillation in patients with low thromboembolic risk

**DOI:** 10.3389/fcvm.2025.1559347

**Published:** 2025-04-24

**Authors:** Meng Wang, Wei Du, Ya-lan Fei, Hao Yang, Qing-shan Dong, Xian-jin Li, Shi-jie Li, Ru-xing Wang, Bing Han

**Affiliations:** ^1^ Xuzhou Clinical College of Xuzhou Medical University, Division of Cardiology, Xuzhou Central Hospital, Xuzhou, Jiangsu, China; ^2^ Division of Cardiology, Wuxi People's Hospital Affiliated to Nanjing Medical University, Wuxi, Jiangsu, China

**Keywords:** atrial fibrillation, anticoagulation, catheter ablation, silent cerebral embolism, low thromboembolic risk

## Abstract

**Objective:**

The aim of this study was to investigate the impact of preprocedural anticoagulation on the incidence of silent cerebral embolisms (SCEs) assessed by magnetic resonance imaging (MRI) after catheter ablation of atrial fibrillation (AF) in patients with low thromboembolic risk.

**Methods and results:**

A total of 141 patients with AF who were identified with low thromboembolic risk based on CHA_2_DS_2_-VASc score (0 or 1 for males and 1 or 2 for females) were enrolled in this study. According to whether or not oral anticoagulants (OACs) had been administered for more than 3 weeks prior to the procedure, patients were divided into the anticoagulation group (*n* = 49) and the non-anticoagulation group (*n* = 92). Pulmonary veins were isolated by utilizing irrigated-tip ablation catheters under the guidance of the Carto system. A cerebral MRI was performed 24 to 48 h after ablation to detect any new-onset SCEs. The incidences of SCEs were compared between the two groups. SCEs were detected in 25 (17.7%) patients. The incidence of SCEs was significantly higher in the non-anticoagulation group compared with the anticoagulation group [22/92 [23.9%] vs. 3/49 [6.1%], *P* = 0.002]. Multivariate logistic regression analysis showed that the preprocedural application of OACs for more than 3 weeks was the only independent protective factor of SCEs after AF ablation.

**Conclusion:**

AF ablation carried a substantial risk of SCEs even in patients with low thromboembolic risk. Preprocedural anticoagulation for more than 3 weeks can significantly reduce the incidence of SCEs after ablation in AF patients.

## Introduction

Atrial fibrillation (AF) is one of the most prevalent cardiac arrhythmias and an important cause of stroke (1, 2). Catheter ablation has evolved into an effective therapeutic strategy for patients with symptomatic AF (3). Cerebral embolism is one of the most crucial complications following AF ablation. Although symptomatic strokes are rare (4), silent cerebral embolisms (SCEs) are relatively common, ranging from 10% to 40% in different reports (5–8). SCEs have been verified to cause cognitive impairment and dementia potentially and should warrant cautious attention (9, 10).

Studies have shown that an uninterrupted periprocedural anticoagulation strategy can reduce strokes and SCEs (11–13). Accordingly, the guidelines recommend continuous anticoagulant therapy before ablation (14). On the other hand, some studies have confirmed that electroconversion was safe following thrombus exclusion using transesophageal echocardiography (TEE) in patients with AF who have not received conventional anticoagulant treatment for over three weeks (15, 16). Moreover, other studies have demonstrated that catheter ablation without standardized use of oral anticoagulants (OACs) does not increase the incidence of thromboembolism for patients with low thromboembolic risk (17). Therefore, the guidelines also suggest using TEE to exclude left atrial (LA) thrombus as an alternative to conventional anticoagulation, especially for low-risk patients (18). However, there is currently insufficient research to determine whether the absence of receiving standardized anticoagulant therapy prior to ablation procedures can increase the occurrences of SCEs in low-risk AF patients. This study aims to investigate the impact of continuous use of OACs before AF ablation on the incidences of postprocedural SCEs in patients with a CHA2DS2-VASC score of 0–1 for males and 1–2 for females.

## Materials and methods

### Patient characteristics

**Figure 1:**
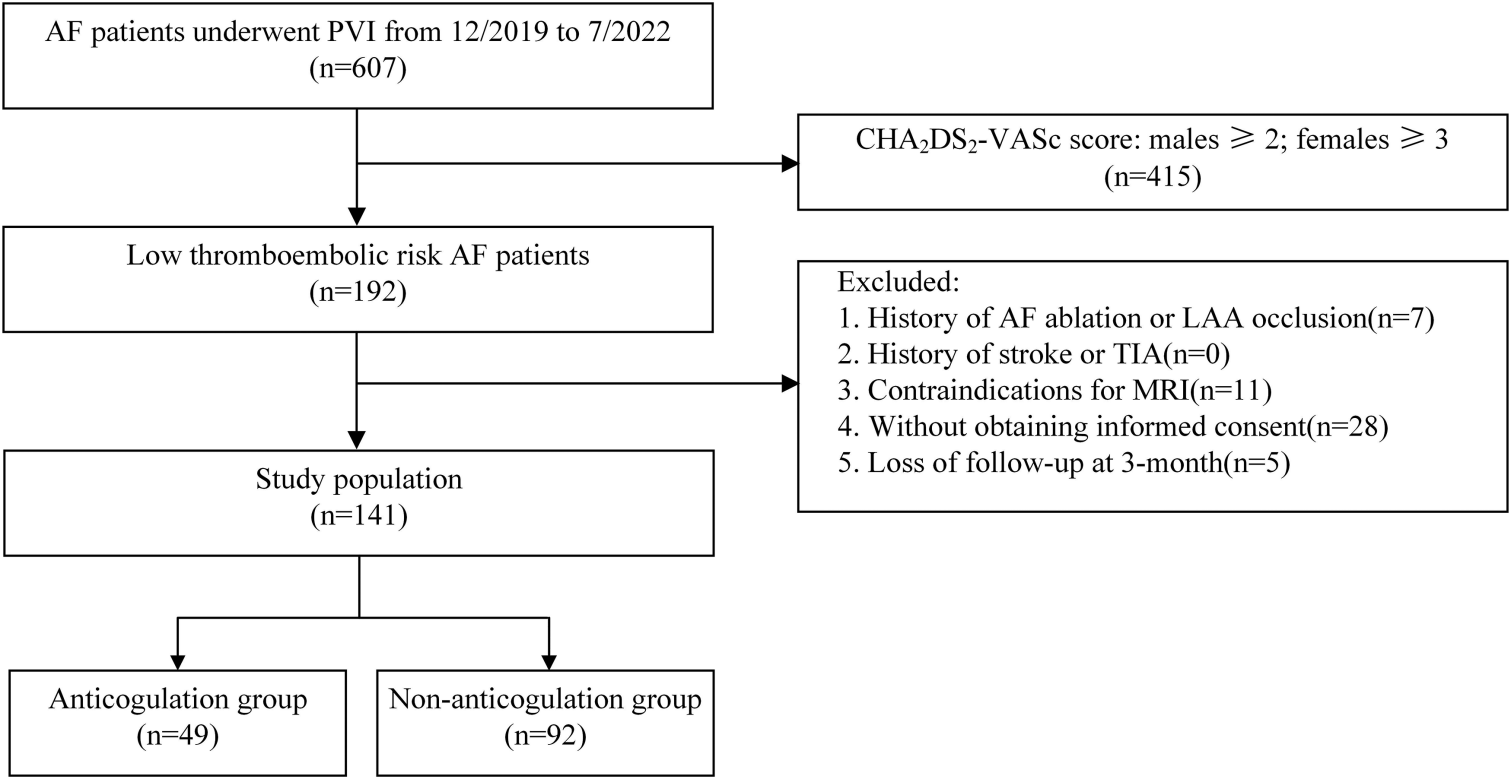
Research flowchart.

AF patients with low thromboembolic risk who underwent their first-time catheter ablation in Xuzhou Central Hospital from December 2019 to July 2022 were enrolled in this study. The inclusion criteria were as follows: documented paroxysmal or persistent symptomatic non-valvular AF, aged between 18 and 80 years, with CHA_2_DS_2_-VASc score (excluding female sex) of 0 or 1. The exclusion criteria included: (1) history of AF catheter ablation or percutaneous left atrial appendage (LAA) occlusion; (2) history of stroke or transient ischemic attacks (TIA); (3) existence of neurological impairment or intracardiac thrombus; (3) contraindications for magnetic resonance imaging (MRI) examination: the presence of cardiac pacemaker, implantable cardioverter defibrillator, or other metal implants that are not compatible with MRI, or severe claustrophobia. Patients who received new oral anticoagulants (NOACs) or warfarin for more than 3 weeks prior to ablation were included in the anticoagulation group. In this group of patients, OACs were uninterruptedly administered on the day of the procedure. Patients not receiving preprocedural anticoagulation were included in the non-anticoagulation group (Figure 1). Antiarrhythmic medications were discontinued at least five half-lives before the procedure. There was no pharmacological or electrical cardioversion in 1 month before PVI in all enrolled patients. Informed consent was signed by all participants prior to study enrollment. The study protocol was reviewed and approved by the ethics committees of Xuzhou Central Hospital.

### Electrophysiological study and catheter ablation

TEE or contrast-enhanced computed tomography (CT) was performed on each patient within 24 h before procedure to confirm the absence of LA thrombus. The ablation protocol was the same as we previously reported (19). Local anaesthesia were administered for all procedures. After double transseptal punctures, two 8.5F SL1 sheaths (St. Jude Medical, St. Paul, MN) were advanced into the LA. A circular mapping catheter (Lasso, Biosense-Webster, Diamond Bar, CA) was introduced through a transseptal sheath to record pulmonary vein (PV) potentials. An irrigated contact force–sensing catheter (ThermoCool SmartTouch, Biosense Webster, Diamond Bar, CA) was used for mapping and ablation. The ablation procedure was performed under the guidance of an electro-anatomic mapping system (CARTO 3, Biosense Webster, Irvine, CA). For all patients, PV isolation (PVI) was the primary ablation strategy. Linear point-by-point lesions were placed in the PV antra with the endpoint of electrical isolation. Radiofrequency energy was delivered continuously while moving the catheter tip every 20–30 s, with a maximum power of 30 W on the posterior wall and 35 W on the anterior wall. The temperature of the radiofrequency energy was limited to 43°C, with irrigation rates of 17 to 30 ml/min. If AF persisted after complete PVI, sinus rhythm (SR) was restored by electrical cardioversion. PV-LA conduction block was confirmed again during SR.

### Anticoagulation during procedure

Baseline activated clotting time (ACT) was measured at the beginning of the procedure. A bolus of unfractionated heparin was injected immediately after the transseptal puncture. Initial heparin dosing was determined according to the baseline ACT and the patient's body weight. ACT was monitored every 20–30 min during the procedure. The target ACT value was ≥300 s in all patients. Once ACT was less than 300 s, a supplemental heparin bolus was given to keep ACT between 300 and 350 s. The total amount of heparin consumption and the mean ACT during the whole procedure were calculated.

### Cerebral magnetic resonance imaging

All patients underwent cerebral MRI at 3.0 Tesla (Philips Medical Systems) 24–48 h after the ablation procedure. Acute SCEs were defined as focal hyperintense lesions on diffusion-weighted (DW) imaging with corresponding hypointense lesions on the apparent diffusion coefficient (ADC) map in a typical vascular pattern, while patients had no neurological disorders. Imaging data were analyzed by two radiologists with at least two years of work experience and blinded to the patient's relevant clinical data. Neurological physical examinations were performed by the physicians both before and after the ablation procedure.

### Postablation care and follow-up

All patients were required to take OACs for at least 2 months after the procedure. Antiarrhythmic drugs were also given during the first 2–3 months. All patients received regular telephone and clinic follow-up to confirm whether there were complications or arrhythmia recurrences.

### Statistical analysis

SPSS software (Version 23.0; IBM, Armnok, NY, USA) was used for statistical analysis, and the difference was considered statistically significant at *P* value < 0.05. Variables in normal distribution were presented as mean ± SD and analyzed via *Student's t*-test, while the non-normally distributed variables were presented as median (interquartile range) and analyzed via *Mann–Whitney U*-test. Categorical variables were presented as numbers (percentages) and compared using the *χ*^2^ test. Univariate and multivariate logistical regression analyses were performed to evaluate the predictors of SCEs. Factors with *P*-values of <0.05 in the univariate analysis were included in a multivariate logistic regression model to identify independent predictors.

## Results

### Patient baseline characteristics and procedural variables

A total of 141 AF patients were enrolled in this study. Of these, 49 patients belonged to the anticoagulation group and 92 to the non-anticoagulation group. There were no significant differences between the two groups at the baseline characteristics (Table 1). With regard to the procedural variables, the ACT before the first heparin bolus was significantly longer, and the total amount of heparin consumption was less in the anticoagulation group than in the non-anticoagulation group. Whereas the procedure time, the mean ACT and the proportion of receiving electrical cardioversion were equivalent in both groups (Table 2).

**Table 1:** Baseline demographic and clinical characteristics of the study population.

Variables	All subjects (*n* = 141)	AG (*n* = 49)	NAG (*n* = 92)	*P*-value
Age (y)	58.7 ± 8.9	59.8 ± 8.0	58.0 ± 9.3	0.265
Male [*n* (%)]	92 (65.2)	30 (61.2)	62 (67.4)	0.464
BMI (kg/m^2^)	25.7 ± 3.3	26.2 ± 3.4	25.4 ± 3.2	0.183
AF duration (month)	15 (5, 48)	15 (5, 48)	14 (4, 48)	0.551
Paroxysmal AF [*n* (%)]	86 (61.0)	27 (55.1)	59 (64.1)	0.295
CHA_2_DS_2_-VA score = 0 [*n* (%)]	55 (39)	19 (38.8)	36 (39.1)	0.967
HAS-BLED score	1 (0, 1)	1 (0, 1)	0 (0, 1)	0.172
Hypertension [*n* (%)]	40 (28.4)	18 (36.7)	22 (23.9)	0.108
Diabetes [*n* (%)]	8 (5.7)	2 (4.1)	6 (6.5)	0.551
Dyslipidemia [*n* (%)]	21 (14.9)	5 (10.2)	16 (17.4)	0.254
Coronary artery disease [*n* (%)]	3 (2.1)	1 (2)	2 (2.2)	0.958
eGFR (ml·min^−1^·1.73 m^−2^)	101.4 ± 12.5	103.3 ± 11.6	100.3 ± 12.9	0.179
LAD (mm)	35.3 ± 6.5	36.6 ± 6.7	34.6 ± 6.3	0.08
LVEDD (mm)	48.0 ± 6.2	47.6 ± 8.0	48.2 ± 5.0	0.556
LVEF (%)	56.5 ± 3.7	56.7 ± 1.8	56.4 ± 4.4	0.609
TEE pr-eprocedure[*n* (%)]	72 (51.1)	26 (53.1)	46 (50)	0.729

AG, anticoagulation group; NAG, non-anticoagulation group; BMI, body mass index; AF, atrial fibrillation; eGFR, estimate glomerular filtration rate; LAD, left atrial diameter; LVEDD, left ventricular end-diastolic dimension; LVEF, left ventricular ejection fraction, TEE, transesophageal echocardiography.

**Table 2:** Comparison of procedural variables between the anticoagulation group and non-anticoagulation group.

Variables	All subjects (*n* = 141)	AG (*n* = 49)	NAG (*n* = 92)	*P*-value
Procedure time (min)	126.4 ± 43.6	132.9 ± 48.4	122.9 ± 40.6	0.193
Baseline ACT (s)	137.1 ± 30.0	154.2 ± 27.7	128.0 ± 27.2	<0.001
Total heparin dose (U)	10437.6 ± 2660.6	9967.4 ± 2460.4	10847.8 ± 2484.7	0.012
Mean ACT (s)	284.13 ± 26.0	286.66 ± 26.2	282.78 ± 25.9	0.4
PVI with additional ablation [*n* (%)]	18 (12.8)	8 (16.3)	10 (10.9)	0.355
Cardioversion [*n* (%)]	48 (34.0)	20 (40.8)	28 (30.4)	0.215

AG, anticoagulation group; NAG, non-anticoagulation group; ACT, activated clotting time; PVI, pulmonary vein isolation.

### The incidence of SCEs and other complications after AF ablation

Bleeding events occurred in two patients of the anticoagulation group, including one case of gastrointestinal bleeding and another case of arteriovenous fistula, while two patients experienced inguinal hematomas in the non-anticoagulation group. All patients with bleeding events remained hemodynamically stable and did not require surgical intervention. One 37-year-old male patient with the CHA2DS2-VASc score of 1 in the non-anticoagulation group experienced a symptomatic stroke in the supply area of the right medial cerebral artery, which caused persistent weakness and difficulty in moving the left limbs the second day after the procedure. The total occurrences of complications were similar between the two groups (Table 3). New on-set SCEs were detected by MRI in 25 (17.7%) patients overall after the procedure. Most lesions were solitary and mainly located in the lobes of the cortex, including frontal lobe, parietal lobe, and occipital lobe. The prevalences between the bilateral hemispheres were comparable (Table 4). The incidence of SCEs was significantly higher in the non-anticoagulation group compared to the anticoagulation group (23.9% vs. 6.1%, *P* = 0.002) (Table 3).

**Table 3:** Complications of the anticoagulation group and non-anticoagulation group.

Complications	All subjects (*n* = 141)	AG (*n* = 49)	NAG (*n* = 92)	*P*-value
All-cause death [*n* (%)]	0	0	0	
Bleeding complications [*n* (%)]	4 (2.8)	2 (4.1)	2 (2.2)	0.516
thromboembolic complications [*n* (%)]	1 (0.7)	0	1 (1.1)	0.464
SCEs [*n* (%)]	25 (17.7)	3 (6.1)	22 (23.9)	0.002

The data are presented as *n* (%). AG, anticoagulation group; NAG, non-anticoagulation group; SCE, silent cerebral embolism.

**Table 4:** Locations and numbers of SCEs.

Case	Group	Numbers	Locations
1	NAG	1	left frontal lobe
2	NAG	1	left frontal lobe
3	NAG	1	left parietal lobe
4	NAG	1	left parietal lobe
5	NAG	1	left parietal lobe
6	NAG	1	left parietal lobe
7	NAG	1	left occipital lobe
8	NAG	1	left cerebellum
9	NAG	1	left cerebellum
10	NAG	2	left anterior horn of lateral ventricle
11	NAG	1	right frontal lobe
12	NAG	2	right frontal lobe
13	NAG	3	right parietal lobe
14	NAG	1	right parietal lobe
15	NAG	1	right parietal lobe
16	NAG	2	right occipital lobe
17	NAG	1	right occipital lobe
18	NAG	1	right parietal lobe, right occipital lobe
19	NAG	1	right occipital lobe, right fibrous crown
20	NAG	1	right caudatum
21	NAG	1	left frontal lobe, right insula
22	NAG	1	left/right frontal lobe, right fibrous crown
23	AG	2	left/right cerebellum
24	AG	1	right cerebellum
25	AG	2	right cerebellum, left posterior horn of lateral ventricle

AG, anticoagulation group; NAG, non-anticoagulation group.

### Comparisons of clinical and procedural characteristics between patients with and without SCEs

Patients with SCEs had longer AF durations and lower left ventricular ejection fraction (LVEF) compared to those without SCEs. There were no differences in the other characteristics. The details were shown in Table 5.

**Table 5:** Characteristics of patients with SCEs and without SCEs.

Variables	SCE group (*n* = 25)	No SCE group (*n* = 116)	*P*-value
Age (y)	59.7 ± 8.5	58.4 ± 9.0	0.508
Male [*n* (%)]	15 (60.0)	77 (66.4)	0.545
BMI(kg/m^2^)	26.7 ± 3.6	25.5 ± 3.2	0.098
AF duration (month)	39 (12, 78)	13 (3, 38)	0.011
Paroxysmal AF [*n* (%)]	17 (68.0)	69 (59.5)	0.428
CHA_2_DS_2_-VA score = 0 [*n* (%)]	10 (40)	45 (38.8)	0.911
HAS-BLED score	1 (0, 1)	1 (0, 1)	0.922
Hypertension [*n* (%)]	5 (20.0)	35 (30.2)	0.308
Diabetes [*n* (%)]	1 (4.0)	7 (6.0)	0.691
Dyslipidemia [*n* (%)]	4 (16.0)	17 (14.7)	0.864
Coronary artery disease [*n* (%)]	1 (4.0)	2 (1.7)	0.474
eGFR（ml·min^−1^·1.73 m^−2^）	99.0 ± 8.6	101.9 ± 13.1	0.251
LAD (mm)	37.9 ± 8.0	34.7 ± 6.0	0.066
LVEDD (mm)	49.9 ± 4.8	47.6 ± 6.4	0.081
LVEF (%)	54.0 ± 6.1	57.0 ± 2.7	0.022
TEE pr-eprocedure[*n* (%)]	12 (48)	60 (51.7)	0.735
Anticoagulation >3 weeks [*n* (%)]	3 (12.0)	46 (39.7)	0.008
Procedure time (min)	129.1 ± 39.9	125.8 ± 35.4	0.428
Baseline ACT (s)	134.9 ± 31.8	137.6 ± 29.7	0.688
Total heparin dose (U)	10700.0 ± 2286.7	10381.0 ± 2740.0	0.588
Mean ACT (s)	276.97 ± 20.6	285.67 ± 26.8	0.129
PVI with additional ablation [*n* (%)]	3 (12)	15 (12.9)	0.899
Cardioversion[*n* (%)]	8 (32.0)	40 (35.4)	0.812

SCE, silent cerebral embolism; BMI, body mass index; AF, atrial fibrillation; eGFR, estimate glomerular filtration rate; LAD, left atrial diameter; LVEDD, left ventricular end-diastolic dimension; LVEF, left ventricular ejection fraction; ACT, activated clotting time; PVI, pulmonary vein isolation; TEE, transesophageal echocardiography.

### Predictors of SCEs after AF ablation

Univariate logistic regression analysis revealed that left atrial diameter (LAD), LVEF, and whether receiving anticoagulation prior to the procedure were associated with the occurrences of SCEs. In a multivariate regression analysis, receiving anticoagulation for more than 3 weeks prior to the procedure was identified as the only independent protective factor for SCEs [odds ratio [*OR*] 0.185, 95% confidence interval [*CI*] 0.049–0.693, *P* = 0.012] (Table 6).

**Table 6:** Univariate and multivariate analysis for predictors of SCEs.

Variables	Univariate analysis			Multivariate analysis		
	OR	95%CI	*P*	OR	95%CI	*P*
Age	1.017	0.967–1.07	0.506			
Male	0.760	0.313–1.846	0.544			
BMI	1.120	0.979–1.281	0.100			
AF duration	1.005	0.992–1.012	0.143			
Paroxysmal AF	0.691	0.276–1.731	0.430			
CHA_2_DS_2_-VA score	0.951	0.393–2.299	0.911			
HAS-BLED score	0.972	0.450–2.099	0.942			
Hypertension	0.579	0.201–1.665	0.310			
Diabetes	0.649	0.076–5.522	0.692			
Dyslipidemia	1.109	0.339–3.634	0.864			
Coronary artery disease	2.375	0.207–27.262	0.487			
eGFR	0.982	0.949–1.016	0.290			
LAD	1.080	1.008–1.156	0.028	1.063	0.977–1.156	0.156
LVEDD	1.090	0.995–1.194	0.065			
LVEF	0.843	0.755–0.941	0.002	0.883	0.777–1.003	0.056
TEE pr-eprocedure	1.161	0.489–2.757	0.736			
Anticoagulation >3 weeks	0.208	0.059–0.733	0.015	0.185	0.049–0.693	0.012
Procedure time	1.005	0.992–1.019	0.424			
Baseline ACT	0.997	0.982–1.012	0.685			
Total heparin dose	1.000	1.000–1.000	0.586			
Mean ACT	0.986	0.969–1.004	0.120			
PVI with additional ablation	1.089	0.290–4.088	0.899			
Cardioversion	0.894	0.355–2.251	0.812			

SCE, silent cerebral embolism; BMI, body mass index; AF, atrial fibrillation; eGFR, estimate glomerular filtration rate; LAD, left atrial diameter; LVEDD, left ventricular end-diastolic dimension; LVEF, left ventricular ejection fraction; ACT, activated clotting time; PVI, pulmonary vein isolation; TEE, transesophageal echocardiography.

## Discussion

The main findings of our study are as follows: (1) 17.7% of AF patients with CHA2DS2-VASc score (excluding female sex) of 0 or 1 undergoing PVI had newly visible SCEs on post-procedural MRI; (2) In these patients, anticoagulation therapy for more than 3 weeks before the procedure was associated with a lower incidence of SCEs.

The CHA2DS2-VASc score has been shown to reliably predict stroke risk in patients with AF and recommended for determining whether oral anticoagulation is warranted (20, 21). Patients with a CHA2DS2-VASc score of 0–1 (excluding female sex) are judged to have a low to intermediate risk of stroke. Although current guidelines do not mandate anticoagulation treatment for these patients (18), there still exists controversy on whether anticoagulation is necessary, and conflicting results have been obtained from various studies. Mobley et al.'s research indicated that AF patients with low CHA2DS2-VASc score are still at a substantially increased risk of thromboembolic events (22). Friberg et al. (23) reported that AF patients with a CHA2DS2-VASc score of 1 have a relatively low risk of ischemic stroke, and these patients do not benefit from routine OAC administration. An observational study by Komen et al. (24) suggested that NOACs treatment might be associated with positive net clinical benefits for patients at low risk for stroke (one non-sex-related CHA2DS2-VASc point). It is important to note that the CHA2DS2-VASc score has certain limitations as it does not consider many factors related to stroke risk, such as duration and load of AF, LA size, renal function, coagulation parameters, etc. (25, 26). Therefore, for patients judged to be at low risk of stroke based on the CHA2DS2-VASc score, a more comprehensive evaluation should be conducted when deciding whether to receive anticoagulant therapy and further research is needed (27).

Many previous studies have indicated that adequate anticoagulation therapy is highly effective in mitigating the risk of thromboembolic complications after AF ablation (11). There are also studies confirming that uninterrupted periprocedural use of OACs can reduce the occurrence of SCEs (12, 13). Based on these findings, current guidelines recommend using OACs for at least three weeks before the ablation procedure as a Class I indication. Meanwhile, excluding LA thrombus by TEE is also recommended as an alternative strategy, especially for low thromboembolic risk patients (18). However, to the best of our knowledge, there have been no studies focusing on the impact of periprocedural anticoagulation management on postprocedural thromboembolism and SCEs in low-risk patients. This pilot study revealed an association between the occurrence of SCEs and periprocedural use of OACs, suggesting that even for patients with low thromboembolic risk, anticoagulation therapy for at least three weeks before AF ablation may be beneficial.

There are several possible reasons for the occurrence of SCE. Firstly, undetected pre-existing LA/LAA microthrombi could be dislodged during the procedure. Although TEE is considered as the gold standard for diagnosing LA thrombus, its sensitivity does not reach 100%, particularly when LAA cannot be adequately visualized (28). Previous studies have confirmed that LA thrombi detected by intracardiac echocardiography were vanished after ≥4 weeks of continuous direct oral anticoagulants (DOACs) therapy (29), suggesting that preprocedural anticoagulation treatment may reduce the occurrence of SCEs caused by intraprocedural microthrombus dislodgment. Secondly, SCEs might have existed before ablation. Due to the lack of preprocedural MR imaging in this study, the existing SCEs could not be ruled out. Herm et al.'s study (30) found that even in low- to medium-risk patients, the detection rate of SCEs was 8%. Thirdly, the occurrence of SCEs was related to ablation or catheter manipulation. Endothelial damage and coagulation system activation caused by ablation, air embolism occurring during sheath insertion, and gas bubbles forming during ablation were all possible causes of cerebral embolism and SCEs (4, 7). Furthermore, the correlation between intraprocedural ACT value and the occurrence of SCEs has received attention. Wazni et al.'s study (31) displayed that maintaining ACT at 300–350 s for patients undergoing AF ablation could significantly reduce periprocedural embolic events compared to maintaining ACT at 250–300 s. Scaglione et al. (32) reported that there was no SCEs occurred for intraprocedural ACT >320 s. Doi et al. (33) indicated that minimum ACT ≤260 s was an independent predictor of SCEs. In the present study, the average ACT value was 284.13 s, which may contribute to the relatively high incidence of SCEs observed in this cohort. Further research is required to determine whether increasing the ACT value during the procedure will have an impact on the outcomes. Another study conducted by Harada M et al. (34) demonstrated that compared with patients without SCEs, those with SCEs had lower baseline ACT before heparin injection and a longer time to reach optimal ACT. In our study, even though the mean ACT level was equivalent between both groups, the anticoagulation group had a higher baseline ACT at the beginning of the procedure. This may be one of the reasons account for the lower incidence of SCEs in this group.

## Study limitations

This study had several limitations. Firstly, this was only a preliminary experience in a single center with a relatively small sample size limited to Chinese patients. Secondly, it was a non-randomized study. There was a risk of selection bias due to whether taking OACs before ablation was at the discretion of the treating physician. Prospective randomized studies involving larger patient populations are required to further determine the impact of anticoagulation strategies on the occurrence of SCEs associated with AF ablation in patients with low thromboembolic risk.

## Conclusions

Even in patients with low thromboembolic risk, there are still substantial risks of SCEs after AF ablation, and continuous anticoagulation therapies before the procedures are associated with lower incidences of SCEs. These findings suggests additional investigations into clinical decision-making for patients with AF at low thromboembolic risk on future preprocedural anticoagulation strategies.

## Data Availability

The raw data supporting the conclusions of this article will be made available by the authors, without undue reservation.
